# Can FT-Mid-Infrared Spectroscopy of Milk Samples Discriminate Different Dietary Regimens of Sheep Grazing With Restricted Access Time?

**DOI:** 10.3389/fvets.2021.623823

**Published:** 2021-04-08

**Authors:** Giovanni Molle, Andrea Cabiddu, Mauro Decandia, Maria Sitzia, Ignazio Ibba, Valeria Giovanetti, Giuseppe Scanu, Margherita Addis, Marco Caredda

**Affiliations:** ^1^Agris Sardegna, Olmedo, Italy; ^2^Associazione Regionale Allevatori (ARA) della Sardegna, Laboratorio Analisi Latte, Nuraxinieddu, Oristano, Italy

**Keywords:** authentication, fatty acids, pasture, chemometrics, FT-MIR, linear discriminant analysis

## Abstract

Milk obtained from sheep grazing natural pastures and some forage crops may be worth a plus value as compared to milk obtained from stall-fed sheep, due to their apparently higher content of beneficial fatty acids (FAs). Fourier transformed mid-infrared (FT-MIR) analysis of FA can help distinguish milk from different areas and diverse feeding systems. The objective was to discriminate milk from sheep and milk from dairy sheep rotationally grazing Italian ryegrass or berseem clover for 2, 4, or 6 h/day. To test this hypothesis, a data-mining study was undertaken using a database of 1,230 individual milk spectra. Data were elaborated by principal component analysis (PCA) and analyzed by linear discriminant analysis (LDA) with or without the use of genetic algorithm (GA) as a variable selection tool with the primary aim to discriminate grazed forages (grass vs. legume), access time (2, 4, or 6 h/day), grazing day (first vs. last grazing day during the 7-day grazing period), and the milking time (morning vs. afternoon milking). The best-fitting discriminant models of FT-MIR spectra were able to correctly predict 100% of the samples differing for the pasture forage, 91.9% of the samples differing for grazing day, and 97.1% of the samples regarding their milking time. The access time (AT) to pasture was correctly predicted by the model in 60.3% of the samples, and the classification ability was improved to 77.0% when considering only the 2 and 6 h/day classes.

## Introduction

Grazing delivers high-quality ruminant products usually at a lower cost as compared to stall feeding (1). Grazing diets of dairy sheep result in positive effects on nutritional and health value, texture, oxidative stability, and flavor of dairy products (2). Moreover, products from pasture are perceived by consumers as more friendly for the environment and animal welfare than those coming from housed systems.

In Mediterranean dairy sheep production systems, diets only with pastures are rather rare because pasture availability is low, at least for part of the pasture growth cycle. Therefore, part-time grazing (PTG) i.e., a time-restricted allocation of ruminants to pasture is a widespread grazing technique in many areas of dairy sheep production. This technique has several benefits compared to 24-h grazing such as a better balancing of ruminant diet and a higher efficiency and evenness of herbage utilization, due to lower sward damages by animal trampling (3).

The allocation to pasture between 4 and 7 h/day can optimize the intake and performance of the dairy sheep (3–5). Besides, PTG can improve the fatty acid (FA) composition of sheep milk (6), particularly if grazing is postponed to afternoon when the grazed herbage is higher in the beneficial polyunsaturated fatty acid (PFA) (n-3) (7).

A key to authenticate and value the grazing feeding regimens in the supply chain of meat and dairy ruminant is to trace milk back to the feeding system (8). The authentication of feeding regimens can be based on biomarkers such as milk FA (9–11), secondary plant metabolites [terpenoids, n-alkanes, and derivatives of chlorophyll (e.g., phytanic and pristanic acids)], and isotopes (12).

The multivariate analysis of spectra captured by Fourier transformed mid-infrared (FT-MIR) spectroscopy has the potential to trace the feeding regimens of cows since spectra contain information that goes beyond that resulting from the analyses of biomarkers (13). Moreover, these methods, if properly calibrated and validated, open up new avenues for the implementation of authentication technology in the dairy industry.

Despite the growing body of knowledge on biomarkers and the development of rapid, low-cost analytical techniques and associated chemometrics that are able to discriminate the feeding regimens of ruminants, tracing of dairy sheep supply chains is still in its infancy.

This paper is an outcome of a wider research program undertaken at Agris Sardegna between 2013 and 2016 for evaluating the impact of PTG on their ingestive behavior and milk production of dairy ewes (3, 4, 14, 15).

This study aims at evaluating the ability of FT-MIR spectra to authenticate individual milk sourced from dairy sheep submitted to PTG at different access time (AT) to different forage crops: a grass (Italian ryegrass, *Loliumitalicum*, Lam) and a legume (berseem clover, *Trifolium alexandrinum* L.).

Genetic algorithms (GAs) were successfully used by our laboratories to select the informative variables for the estimation of the sheep milk fatty acids in FT-MIR spectroscopy (16) and in the selection of the FA and the FT-MIR spectral regions that are able to trace the geographical origin of sheep milk (17).

The specific objective of this study was to assess the ability of linear discriminant analysis (LDA) with or without the use of GA to discriminate (a) grass vs. legume pastures; (b) AT (2, 4, or 6 h/day), (c) grazing day (first vs. last grazing day during a 7-day grazing period); (d) milking time (morning vs. afternoon). To this aim, in order to set a benchmark for model interpretation, the effects of the factors under study were evaluated using both univariate analysis and multivariate principal component analysis (PCA).

## Materials and Methods

### Pasture and Sheep Feeding

The milk samples were collected in 2013 (Experiment 1, E1) and 2014 (Experiment 2, E2) from Sarda ewes under PTG of grass (G, E1), Italian ryegrass (*Lolium multiflorum* Lam, cultivar Teanna), and a legume (L, E2) berseem clover (*Trifolium alexandrinum* L, cultivar Laura). The experiments were conducted at the Bonassai research station, north-western Sardinia [40° N, 8° E, 32 meters above sea level (m.a.s.l.)] from the end of February to early May in both years (growth period of the pasture). In both studies, 36 mid-lactation Sarda ewes, divided into replicated groups (two groups per treatment) part-time grazed their pasture for an AT of 2 h/day (8:00–10:00), 4 h/day (8:00–12:00), or 6 h/day (8:00–14:00). The pasture plots, divided by electric fences into four subplots, were rotationally grazed, with 7 days of occupation per subplot and a recovery period of 21 days.

The ewes were machine-milked twice daily at 07:00 h and 15:00 h. After morning milking, the groups were carried on a trailer to the plots where they spent the scheduled time. During the remaining daytime, the ewes were kept indoor in separate pens. The ewes were supplemented daily with pellet concentrate (400 g/head, divided into two meals at milking), lupin seed (300 g/head, E1), or whole maize grain (300 g/head, E2) after grazing, and 700 g/head of ryegrass-based hay overnight. The flat supplementation rate was set in order to meet 100% of the energy requirement of the 4 h/day treatment and 100% metabolic protein requirements of the 2 h/day treatment. For details on pasture establishment, animal management, methods adopted, and performance results, the reader can refer to Molle et al. (3) (E1) and Molle et al. (4) (E2). A summary of the average group diet composition and energy intake on the first and last days of the grazing period is given in Table 1.

**Table 1:** Diet composition and energy intake of dairy ewes part-time grazing (PTG) with different access time (AT, h/day) to pastures of Italian ryegrass or berseem clover as measured on the first (day 1) and last day (day 7) of the grazing period of 7 days.

**Forages/trial**	**AT**	**Grazing day**	**Ash**		**EE**		**CP**		**NDF**		**ADF**		**NFC**		**IVDMD**		**Intake NE_**L**_**	
			**Mean**	**SD**	**Mean**	**SD**	**Mean**	**SD**	**Mean**	**SD**	**Mean**	**SD**	**Mean**	**SD**	**Mean**	**SD**	**Mean**	**SD**
Ryegrass/E1	2	1	105	7	34	2	156	9	461	10	247	7	245	17	754	23	2.7	0.3
		7	104	7	32	2	148	7	474	21	254	15	243	20	734	45	2.5	0.4
	4	1	107	10	35	2	154	12	456	12	241	9	249	27	773	32	3.4	0.6
		7	107	11	32	2	141	8	479	27	256	16	241	28	735	59	2.9	0.5
	6	1	107	11	35	2	158	13	452	15	236	10	248	24	775	33	3.8	0.6
		7	111	10	31	2	140	8	485	22	258	15	233	18	726	49	3.0	0.5
Clover/E2	2	1	102	6	41	4	173	12	382	42	229	21	301	33	764	38	3.5	0.5
		7	104	7	39	4	165	6	381	28	232	14	311	27	760	20	3.5	0.5
	4	1	108	3	42	3	181	11	371	23	225	10	298	19	779	24	4.5	0.4
		7	116	14	39	6	169	12	377	25	232	18	299	15	772	24	4.6	0.7
	6	1	106	4	44	4	187	12	360	36	214	15	303	27	792	25	4.5	0.2
		7	112	8	40	7	178	14	363	25	221	15	307	18	781	27	4.4	0.7

*Each mean refer to n = 8 group data; Means and SD*.

*Diet composition expressed as g/kg DM and net energy intake (NE_L_) as Mcal/head day*.

*EE, ether extract; CP, crude protein; NDF, neutral detergent fiber (ash excluded); ADF, acid detergent fiber; NFC, non-fiber-carbohydrates; IVDMD, pepsine–cellulase in vitro dry matter digestibility*.

### Samplings

Milk yield and milk composition were measured on all ewes on day 1 and 7 of each grazing period. Milk samples were assayed for milk fat, protein, and lactose contents (Milkoscan FT+, Foss Electric, Hillerød, Denmark). Excluding the pre-experimental, adaptation period, 632 samples were gathered from March 20, 2013 to April 28, 2013 (E1), and 598 samples were gathered from 11 March 2014 to 22 April 2014 (E2), resulting in a total of 1,230 samples.

### Fourier Transformed Mid-Infrared Spectra

**Figure 1:**
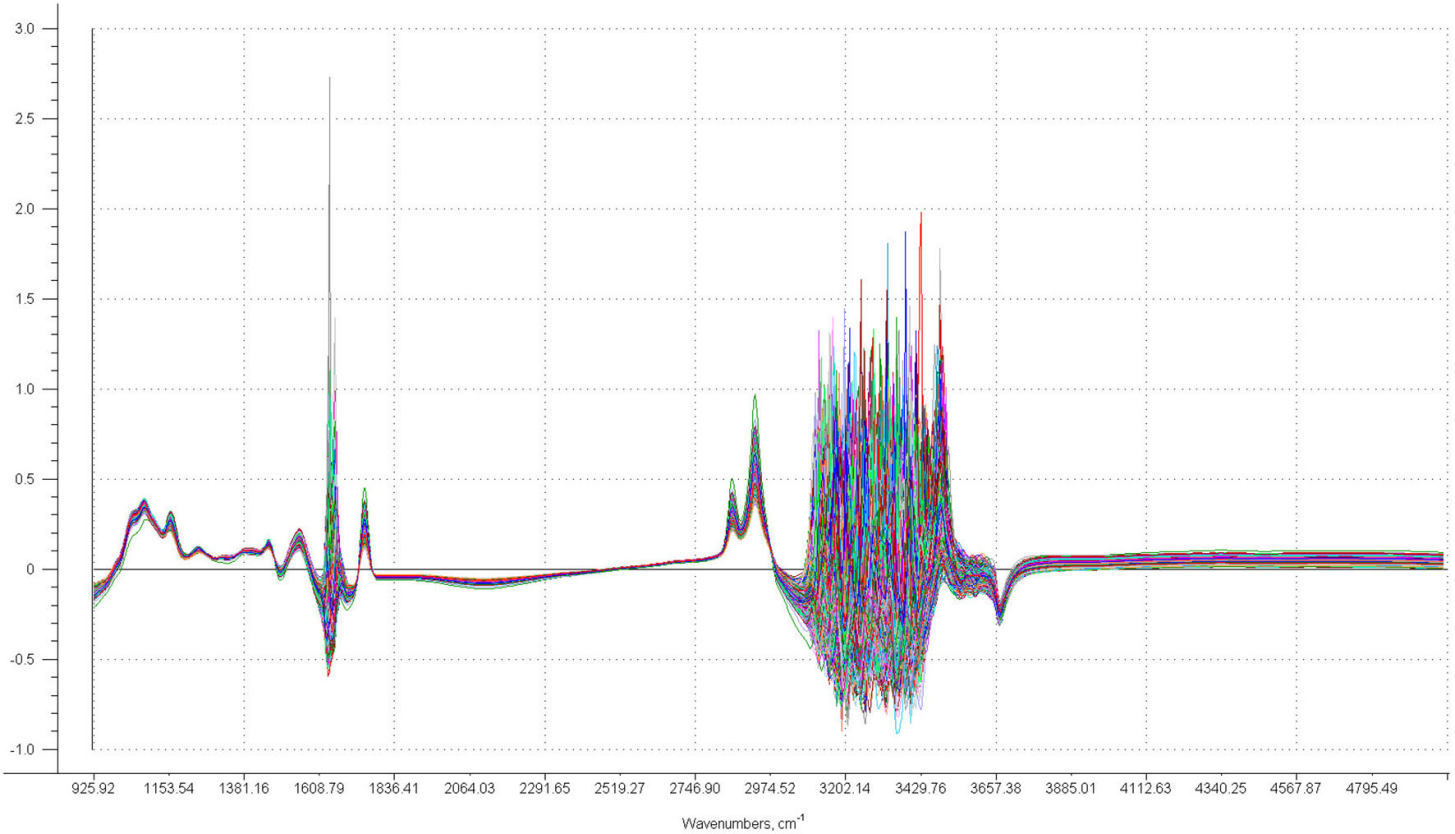
Plot of the overlapped 1,230 milk sample spectra.

Fourier transformed mid-infrared spectra of the sheep milk samples were recorded on a Spectrometer Milkoscan FT6000 (Foss Electric, Hillerød, Denmark) in the spectral region between 925.9 and 5,011.5 cm^−1^. The instrumental resolution was 3.858 cm^−1^, and each spectrum consisted of 1,060 data points. The acquisition of each sample was carried out as duplicate and then averaged. Figure 1 shows the overlapping of the 1,230 milk spectra.

Since the regions from 1608.8 to 1697.5 cm^−1^ and from 3044.0 to 3850.3 cm^−1^ were characterized by a strong instrumental noise and the region from 3850.3 to 5011.5 cm^−1^ was characterized by pure baseline, these regions were not used in the data analysis. Therefore, we considered the regions between 925.9 and 1604.9 cm^−1^ and between 1701.4 and 3040.1 cm^−1^ as “whole spectrum,” totaling 525 spectral variables.

### Principal Component Analysis of the Spectral Data Set

**Figure 2:**
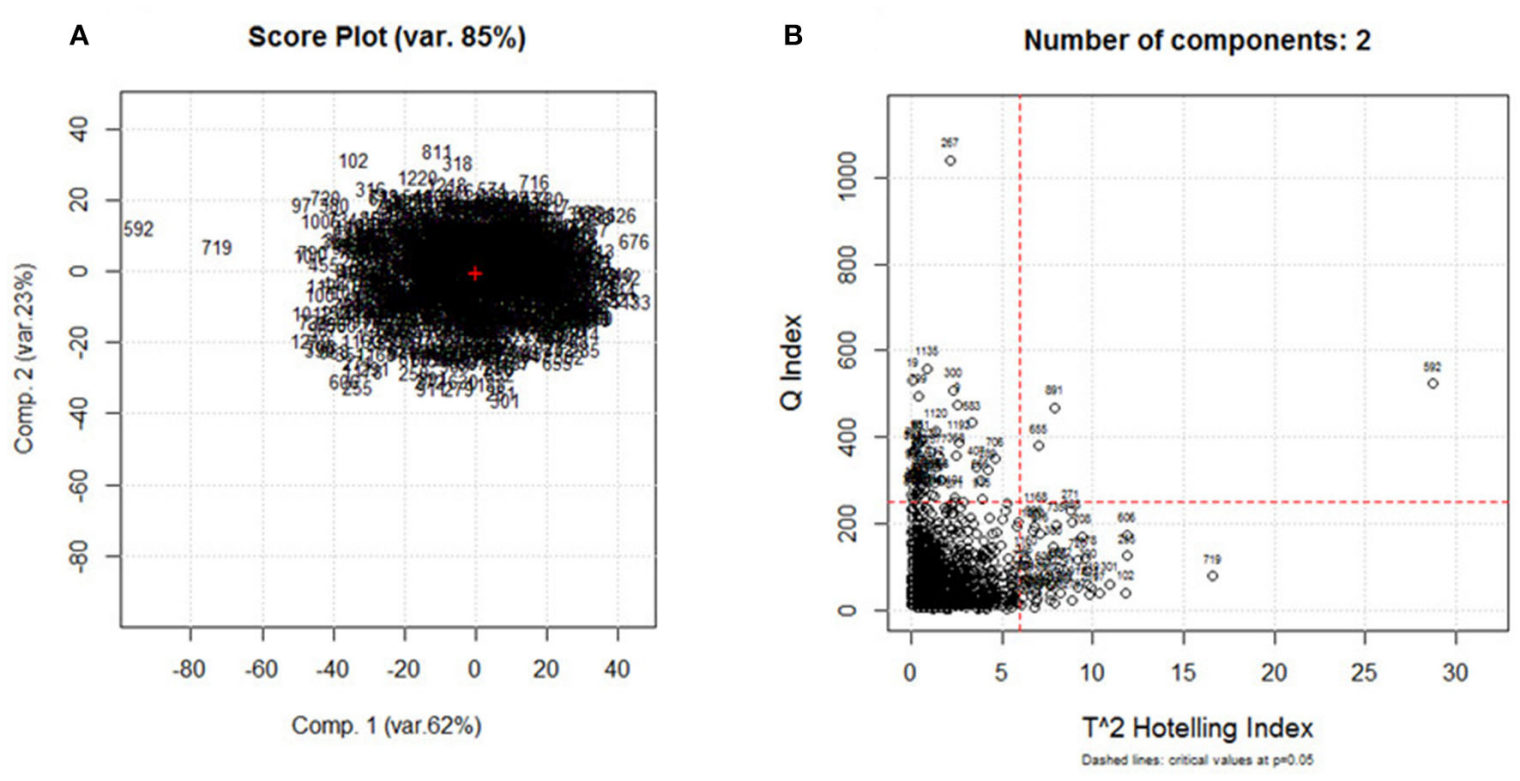
**(A)** Score plot obtained on the principal component analysis (PCA) of the spectral data set (1,230 samples × 525 variables) and **(B)** T^2^ vs. Q diagnostic plot.

Principal component analysis was performed on the database consisting of 1,230 samples and 525 spectral variables. Data were centered and scaled. The obtained score plot (Figure 2A) and diagnostic plot T^2^ vs. Q (Figure 2B) were used to identify possible outliers. Five samples were considered as outliers and removed from the data set; the data now contain 1,225 samples. PCA was run on the chemometric agile tool (CAT) software, developed by the Group of Chemometrics of the Division of Analytical Chemistry of the Italian Chemical Society, freely downloadable from the site, gruppochemiometria.it.

### Prediction of Fatty Acids in Milk by FT-MIR

The fatty acid (FA) composition of the 1,225 milk samples was predicted by FT-MIR spectroscopy, using previously published prediction models (16). The predicted (FAs), expressed as g/100 g fatty acid methyl esters (FAME), include C4:0, C6:0, C8:0, C10:0, C12:0, C14:0, C16:0, C18:0, C18:1 9c, C18:1 11t; C18:2 9c 12c, C18:3 9c 12c 15c, C18:2 9c 11t, and the classes of saturated fatty acids (SFA), unsaturated fatty acids (UFA), monounsaturated fatty acids (MUFA), polyunsaturated fatty acids (PUFA), omega 6 (n-6), and omega 3 (n-3).

### Univariate Analysis and PCA of the FA Profile

The predicted FA database was subjected to a mixed model analysis for repeated measurements considering fixed factors, pasture forage species/trial (grass and legume), AT (2 h/day, 4 h/day, and 6 h/day), grazing day during grazing period (first, day 1 and last, day 7), milking time [morning (M) and afternoon (A)] and all their first- order interactions and the ewe within the treatment group as random factor. Means were compared by Tukey–Kramer *t*-test, when effects were significant at *p* < 0.05. Trends are presented and discussed if *p* < 0.10.

Principal Component Analysis was performed on the milk FA profile to visualize any trend in data. Data were centered and scaled.

### Building of the Discriminant Models

First, the samples were labeled considering four different types of possible categorization, which include (1) the pasture forage, confounded with the supplementation type (named as forage/trial effect); (2) the AT to pasture; (3) the grazing day; (4) the milking time.

As for the AT to pasture, four different sample partitions were performed comparing the following factors: (a) the three categories (i.e., the treatment groups: 2, 4, and 6 h/day); (b) the 2 h/day and 4 h/day samples grouped together in one category against the 6 h/day samples; (c) the 4 h/day and 6 h/day samples grouped together in one category against the 2 h/day samples; (d) the 2 h/day samples against the 6 h/day samples.

Therefore, totally, we performed seven trials; for each one, the samples were randomly divided into a training set and a test set containing about 60 and 40% of the samples, respectively. Table 2 summarizes the different trials and the sample partitioning into two sample sets (training and test).

**Table 2:** Trials and sample subdivision into training and test sets.

**Trial**		**Categories**	**Number of samples**	
			**Training set**	**Test set**
Pasture forage		Grass	393	239
		Legume	341	252
Access time (AT) to pasture	(a)	2 h/day	244	162
		4 h/day	245	165
		6 h/day	245	164
	(b)	(2 and 4) h/day	489	327
		6 h/day	245	164
	(c)	2 h/day	244	162
		(4 and 6) h/day	490	329
	(d)	2 h/day	244	162
		6 h/day	245	164
Grazing day		Day 1	397	262
		Day 7	337	229
Milking time		Afternoon	374	236
		Morning	360	255

Linear discriminant analysis (LDA) was used for discriminating the sheep milk samples based on their respective categories. For each trial, we proceeded as follows. In the first step, we built discriminant models using all FA or only the informative FA selected by GAs as predictors. In the second step, we built discriminant models using FT-MIR spectra as predictors. Different spectral pretreatments, such as the first and second derivatives, standard normal variate (SNV) and multiplicative scatter correction (MSC) were evaluated, finding no improvement of accuracy as compared to non-pretreated spectra. Therefore, we presented only the trials performed using the non-pretreated spectra.

For each discriminant model, calibration was performed by cross-validation (CV), using samples from the training set and validation was run using samples from the test set (prediction of an external set of samples).

When we applied LDA to FT-MIR spectra, different predictors were considered, as LDA cannot use the whole spectra because the number of correlated variables would be too high:

(1) the scores obtained in the PCA of the spectral data set.

To do this, the following procedure was applied:

- principal component analysis of spectra training set;
- projecting the spectra of the test set on the PCA model obtained on the training set;
- use the obtained scores, i.e., those corresponding to the most informative components of the PCA obtained from the training set;

(2) the average of three contiguous wavelengths of each milk spectrum, obtaining a reduction from 525 to 175 spectral variables;
(3) the informative spectral variables selected by applying the GA to the spectral data set. A different selection of wavelengths was done for each trial. Since the efficiency of GA decreases when the number of variables is >200 (18), we applied GA to the averaged spectra of 175 variables. GA procedure was replicated five times in order to achieve a more consistent model. The spectral regions selected in the five runs were then compared and only those selected by the majority of the runs were retained in the final model. The selected variables were then reported on the original spectra composed of 525 variables.

Linear discriminant analysis was run on the CAT software, developed by the Group of Chemometrics of the Division of Analytical Chemistry of the Italian Chemical Society, freely downloadable from the site, gruppochemiometria.it.

## Results

### Univariate and PCA of Predicted FA Profile

The univariate analysis of the predicted FA profile of the 1,225 sheep milk samples showed significant effects of pastures on forage species/trial and milking time on all FAs and their classes (Table 3). In contrast, the AT to pasture had a significant effect only on some short- to medium-chain FA (C6:0, C8:0, C10:0, and C12:0) and n-3 (*p* < 0.050), whereas the grazing day affected all variables with the exception of C12:0 (*p* < 0.073) and UFA. The content of short-chain fatty acids (with the exception of C6:0) and SFA was higher in milk samples from sheep grazing the legumes than the grass pastures. Also, the beneficial FAs (C18:1 11t; C18:2 9c 11t; C18:3 9c 12c 15c), PUFA, and n-3 had higher values in the samples from the legume-based diets. The same beneficial FAs were higher in the samples obtained from the first than the last grazing day and from the morning than the afternoon milking, with the exception of n-3 FA.

**Table 3:** Fatty acid (FA) profile (means expressed as g/100 g fatty acid methyl ester (FAME), mean and standard error of the mean (SEM) of the sheep milk samples as estimated using Fourier transformed mid-infrared (FT-MIR) calibrations.

	**C4**	**C6**	**C8**	**C10**	**C12**	**C14**	**C16**	**C18**	**C18:1 c9**	**C18:1t11**	**C18:2**	**C18:3**	**CLA**	**SFA**	**UFA**	**MUFA**	**PUFA**	**n-6**	**n-3**
**Fo/trial**																			
Grass	3.93	3.67	2.18	6.25	3.54	10.09	24.00	10.42	18.60	1.32	2.31	0.67	0.85	68.33	31.19	26.45	5.89	3.00	1.31
Legume	4.16	3.05	2.62	7.92	4.33	11.13	24.54	9.39	12.90	3.08	1.83	1.04	1.48	70.46	29.75	23.49	7.02	3.09	2.13
**At**																			
2 h/day	4.03	2.77 a	2.30 a	6.74 a	3.78 a	10.42	24.23	10.01	16.30 b	2.14	2.11	0.84	1.13	68.66	30.99	25.44	6.38	3.07	1.68
4 h/day	4.02	2.86 ab	2.40 ab	7.13 ab	3.96 ab	10.69	24.47	9.95	15.70 ab	2.18	2.04	0.84	1.16	69.64	30.15	24.85	6.40	3.03	1.67
6 h/day	4.09	2.95 b	2.49 b	7.38 b	4.08 b	10.72	24.11	9.75	15.24 a	2.28	2.07	0.88	1.19	69.89	30.27	24.61	6.59	3.04	1.81
***Gd***																			
1	4.12	2.93	2.46	7.19	3.96	10.55	23.96	9.84	15.38	2.26	2.04	0.87	1.19	69.07	30.38	24.79	6.57	2.99	1.78
7	3.96	2.79	2.34	6.97	3.91	10.67	24.58	9.98	16.11	2.15	2.11	0.84	1.14	69.72	30.56	25.14	6.34	3.10	1.66
**Mt**																			
Afternoon	3.98	2.75	2.27	6.67	3.77	10.52	24.08	9.99	16.71	2.16	2.12	0.84	1.14	68.55	31.25	25.61	6.37	3.08	1.76
Morning	4.11	2.98	2.53	7.50	4.10	10.70	24.46	9.82	14.79	2.24	2.03	0.87	1.18	70.24	29.70	24.32	6.54	3.01	1.68
Mean	4.06	2.88	2.41	7.10	3.94	10.60	24.25	9.89	15.71	2.16	2.07	0.85	1.15	69.41	30.35	24.88	6.43	3.04	1.71
SEM	0.01	0.01	0.01	0.04	0.02	0.04	0.07	0.04	0.11	0.03	0.01	0.01	0.01	0.15	0.10	0.09	0.03	0.01	0.02
**Effects**, ***p*****<**																			
Fo/trial	0.000	0.000	0.000	0.000	0.000	0.000	0.092	0.000	0.000	0.000	0.000	0.000	0.000	0.000	0.002	0.000	0.000	0.002	0.000
AT	0.662	0.018	0.018	0.007	0.013	0.165	0.665	0.566	0.027	0.332	0.114	0.221	0.405	0.099	0.311	0.207	0.167	0.403	0.051
Graze day	0.000	0.000	0.000	0.000	0.073	0.030	0.000	0.014	0.000	0.002	0.000	0.001	0.002	0.016	0.258	0.002	0.000	0.000	0.000
Mt	0.000	0.000	0.000	0.000	0.000	0.001	0.003	0.002	0.000	0.027	0.000	0.004	0.003	0.000	0.000	0.000	0.000	0.000	0.000
Fo × AT	0.378	0.547	0.190	0.023	0.017	0.030	0.411	0.004	0.141	0.041	0.051	0.024	0.092	0.570	0.203	0.359	0.500	0.682	0.385
At × Gd	0.011	0.331	0.993	0.761	0.402	0.798	0.542	0.118	0.400	0.993	0.865	0.154	0.602	0.895	0.651	0.700	0.639	0.650	0.420
Fo × Gd	<0.0001	0.002	0.015	0.498	0.240	0.603	0.299	<0.0001	0.031	<0.0001	0.601	0.000	0.000	0.001	0.693	0.231	0.197	0.001	0.429
AT × Mt	0.440	0.468	0.585	0.729	0.774	0.958	0.843	0.000	0.007	0.108	0.720	0.181	0.157	0.817	0.093	0.073	0.122	0.074	0.036
Gd × Mt	0.164	0.308	0.878	0.705	0.215	0.001	0.001	0.272	0.321	0.419	0.669	0.030	0.080	0.010	0.585	0.622	0.024	0.418	0.214
Fo × Mt	0.272	0.732	0.240	0.010	0.007	0.116	0.176	0.002	0.000	0.003	0.000	0.969	0.162	0.175	0.002	0.000	0.762	0.000	0.687

*Fo/trial, pasture forage/trial; AT, access time; Gd, grazing day; Mt, milking time*.

On the contrary, C18:2 9c 12c and MUFA had higher values in milk from the grass-fed sheep, afternoon milking, and the last grazing day.

Several interactions between factors affected the FA milk composition (Table 3) such as the one between AT and pasture forage. In fact, extending AT to pasture, the levels of C18:2 9c 11t and C18:3 9c 12c 15 c increased more in the milk of ewes grazing the grass in E1 than the legume in E2 (*p* < 0.05). Another significant interaction was between pasture forage and grazing day: C18:1 11t and C18:2 9c 11t decreased, whereas SFA increased when passing from the first to the last grazing day but only in the milk of the legume-grazing sheep (*p* < 0.05). On the contrary, C18:3 9c 12c 15c decreased during the grazing period only in the milk of the grass-grazing sheep.

Also, the milking time significantly interacted with pasture forage, AT, and grazing days. The morning milking samples had higher values of C18:1 11t than the afternoon samples, mainly in the samples of legume-grazing sheep alone (*p* < 0.01). Moreover, C18:2 9c 11t, C18:3 9c 12c 15c, and PUFA decreased in the morning samples when passing from the first to the last grazing day (*p* < 0.08 for C18:2 9c 11t, *p* < 0.05 for C18:3 9c 12c 15c, and PUFA). It is worth noting that n-3 levels were similar between afternoon and morning milking, only when AT was 6 h/day. With shorter AT, the levels were generally higher in the afternoon than in the morning milking samples.

**Figure 3:**
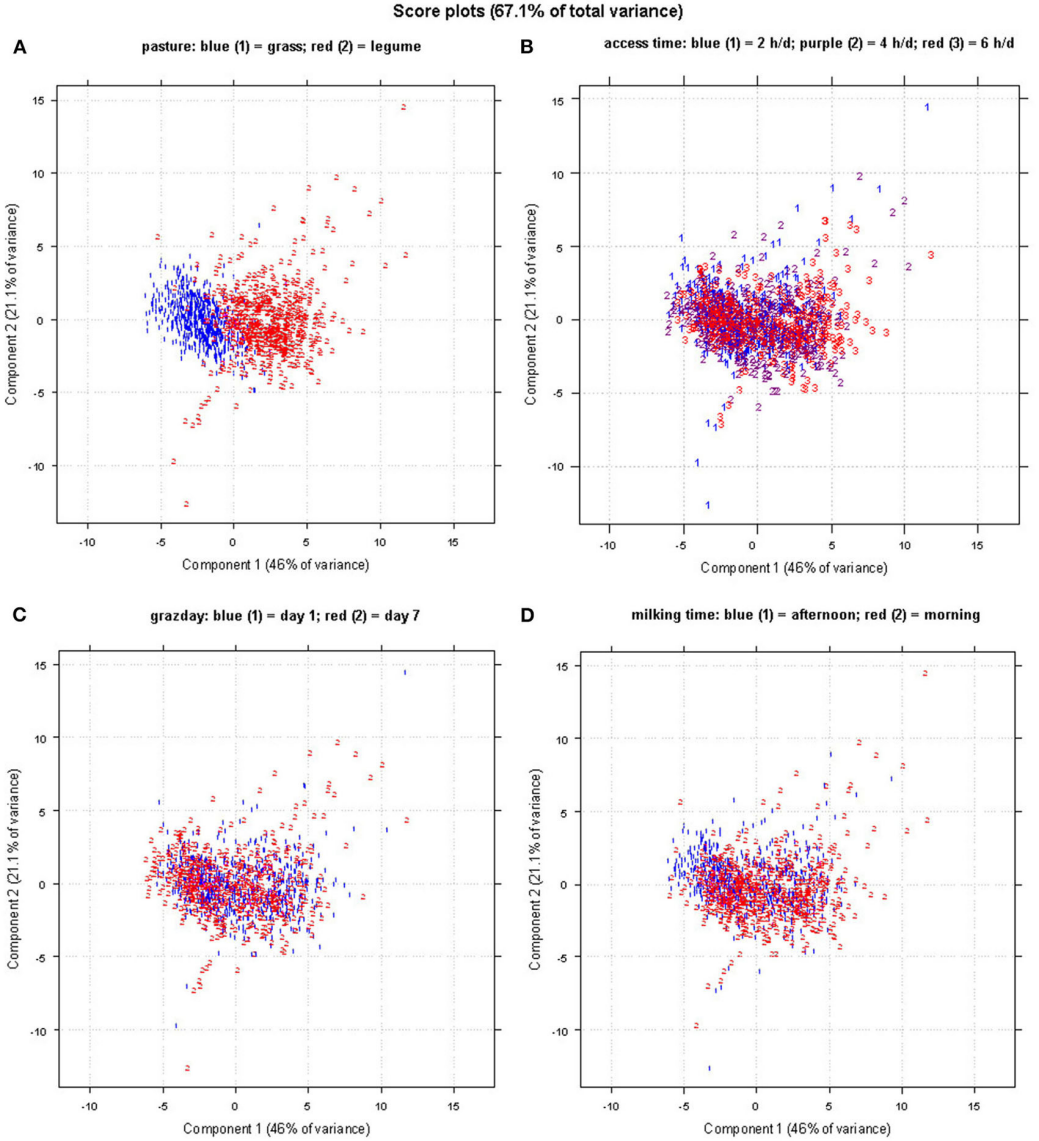
Score plot of the principal component analysis (PCA) obtained using the fatty acid (FA) profile data set; samples are labeled and colored as for the classifications of **(A)** pasture forage, **(B)** access time, **(C)** grazing day, and **(D)** milking time.

**Figure 4:**
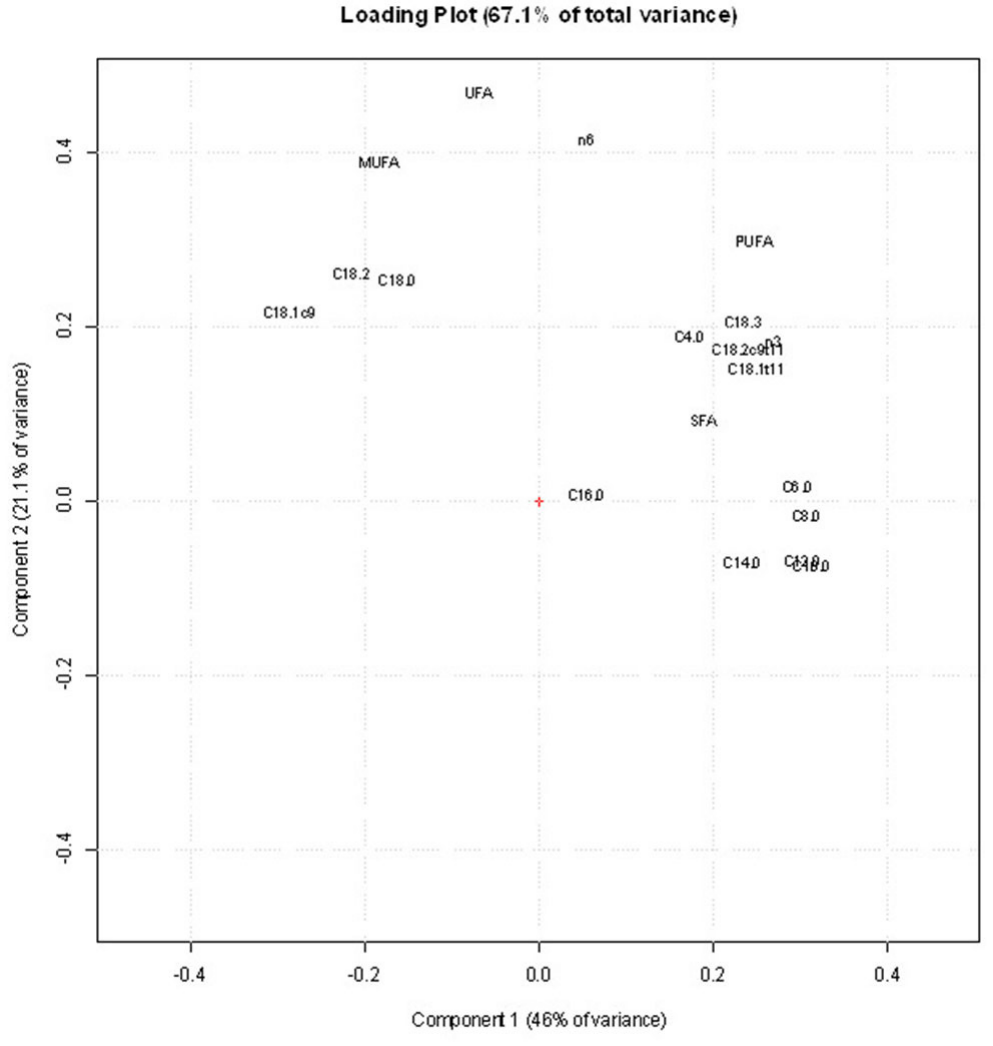
Loading plot of the principal component analysis (PCA) obtained using the fatty acid (FA) profile data set.

Figure 3 shows the score plot of the PCA of the FA data set of 1,225 sheep milk samples with reference to the following four types of categorization: (a) pasture forage/trial (grass and legume); (b) AT to pasture (2, 4, and 6 h/day); (c) grazing days (day 1 and 7); (d) milking times (A and M). The PCA of FA allowed to visually distinguish only the samples differing for the pasture forage/trial (Figure 3A), being the first principal component the axis in which the samples are separated. In the corresponding loading plot (Figure 4), the first component differentiates the samples based on their content of short- and medium-chain FAs (C6:0, C8:0, C10:0, C12:0, and C14:0), SFA, and some UFAs, such as C18:1 11t and C18:2 9c 11t. All these FAs had higher values in the milk samples of the legume-fed ewes (Table 3). Instead, the second principal component describes the variability inside each group of samples, which is mainly due to MUFA, UFA, and n-3. For the other types of categorizations, the visual separation of samples was not possible, even when plotting other principal components.

#### Discriminant Analysis Using the FA Profile as Predictors

Table 4 shows the results of LDA using both the entire FA profile and the selected FAs to predict the different origins of the milk samples.

**Table 4:** Percentage of correct classifications obtained by linear discriminant analysis (LDA) using the fatty acid (FA) profile or the FA selected by genetic algorithm (GA) as predictors.

**Predictors**	**Pasture forage**		**AT**		**AT**		**AT**		**AT**		**Grazing day**		**Milking time**	
	**Grass vs. Legume**		**2 vs. 4 vs. 6**		**(2+4) vs. 6**		**2 vs. (4+6)**		**2 vs. 6**		**Day 1 vs. Day 7**		**Morning vs. Afternoon**	
	**CV**	**Pred**.	**CV**	**Pred**.	**CV**	**Pred**.	**CV**	**Pred**.	**CV**	**Pred**.	**CV**	**Pred**.	**CV**	**Pred**.
FA profile	100	100	47.8	43.8	63.1	57.9	64.0	60.8	67.3	60.8	72.3	74.9	88.6	88.8
FA selected by GA	100	100	50.0	41.9	65.7	59.9	65.1	59.4	68.3	61.4	74.8	73.0	89.0	88.8
Selected FA	C18:1 9c C18:2 9c 12c C18:3 9c 12c 15c C18:2 9c 11t PUFA n-6 n-3		C4:0 C6:0 C8:0 C10:0 C12:0 C16:0 C18:0 C18:1 11t C18:2 9c 12c UFA n-6 n-3		C4:0 C6:0 C8:0 C10:0 C12:0 C14:0 C16:0 C18:0 C18:1 9c C18:1 11t C18:2 9c 12c C18:3 9c 12c 15c C18:2 9c 11t UFA n-6 n-3		C4:0 C6:0 C8:0 C10:0 C12:0 C14:0 C16:0 C18:0 C18:1 9c C18:1 11t C18:3 9c 12c 15c C18:2 9c 11t SFA UFA n-6		C4:0 C6:0 C8:0 C10:0 C12:0 C14:0 C16:0 C18:0 C18:1 9c C18:1 11t C18:2 9c 12c C18:3 9c 12c 15c UFA n-6 n-3		C4:0 C6:0 C8:0 C12:0 C14:0 C18:1 9c C18:1 11t C18:2 9c 12c SFA PUFA n-6 n-3		C6:0 C8:0 C10:0 C14:0 C16:0 C18:1 9c C18:2 9c 12c C18:2 9c 11t SFA UFA PUFA n-6 n-3	
N. selected variables	7		12		16		15		15		12		13	

*AT, access time; CV, cross validation; Pred., prediction; PUFA, polyunsaturated fatty acid; UFA, unsaturated fatty acid; SFA, saturated fatty acid*.

#### Discrimination of the Pasture Forage

When discriminating the pasture forages, the FAs were able to classify 100% of the samples of the training set and to correctly predict 100% of the test set samples. Applying GAs to the FA data set led to a reduction of the number of variables to be used in the prediction model. GA selected only C18:2 9c 12c, C18:3 9c 12c 15c, C18:2 9c 11t, PUFA, n-6, and n-3, maintaining the same accuracy of the model built using all the variables as predictors.

#### Discrimination of the AT to Pasture

Using the whole FA profile as the predictor, only 47.8% of the samples of the training set and 43.8% of the test set were correctly classified in the three categories, 2, 4, and 6 h/day. As can be seen in Table 4, an improvement was obtained grouping the 4 h/day with either 2 h/day samples or 6 h/day samples and comparing these new categories with the remaining ones (6 or 2 h/day, respectively). A further slight improvement was obtained when the LDA of FA was used to discriminate the 2 h/day from the 6 h/day samples, without considering the 4 h/day samples. In this case, 67.3% samples from the training set and 60.8% samples from the test set were correctly predicted.

Applying the GA allowed for a reduction of the number of variables in all the models. In the LDA of the three categories (2, 4, and 6 h/day), GA selected all individual SFAs (with the exception of C14:0), together with C18:1 11t, C18:2 9c 12c, and the classes, UFA n-6, and n-3. The resulting discriminant model correctly predicted 50.0 and 41.9% of the training and test set samples, respectively. As in the case of LDA, the LDA-GA of FA profile achieved slightly better discrimination accuracies when grouping the samples in order to compare only two categories (Table 4). The best result was obtained from the discrimination of the 2 h/day from the 6 h/day samples. The GA selected all the individual FAs (with the exception of C18:2 9c 11t), and the classes UFA, n-6, and n-3. The model built with the selected variables as predictors correctly classified 68.3% of the training set samples and 61.4% of the test set samples.

#### Discrimination of the Grazing Day

The model built using the whole FA profile as a predictor for the discrimination of the grazing day correctly predicted 72.3 and 74.9% of the training samples and of the test set samples, respectively. The results were similar when building the model using the GA-selected variables, with 74.8% of the training set samples and 73.0% of the test set samples correctly classified. The selected variables include, C4:0, C6:0, C8:0, C12:0, C14:0, C18:1 9c, C18:1 11t, C18:2 9c 12c, SFA, PUFA, n-6, and n-3.

#### Discrimination of the Milking Time

The model built with all the FA variables correctly predicted the milking time origin in 88.6% and 88.8% of the training and test set samples, respectively. GA selected C6:0, C8:0, C10:0, C14:0, C16:0, C18:1 9c, C18:2 9c 12c, C18:2 9c 11t, SFA, UFA, PUFA, n-6, and n-3, and the model built using these variables correctly predicted 89.0% of the training set samples and 88.8% of the test set samples.

### Discriminant Analysis Using the FT-MIR Spectra as Predictors

**Figure 5:**
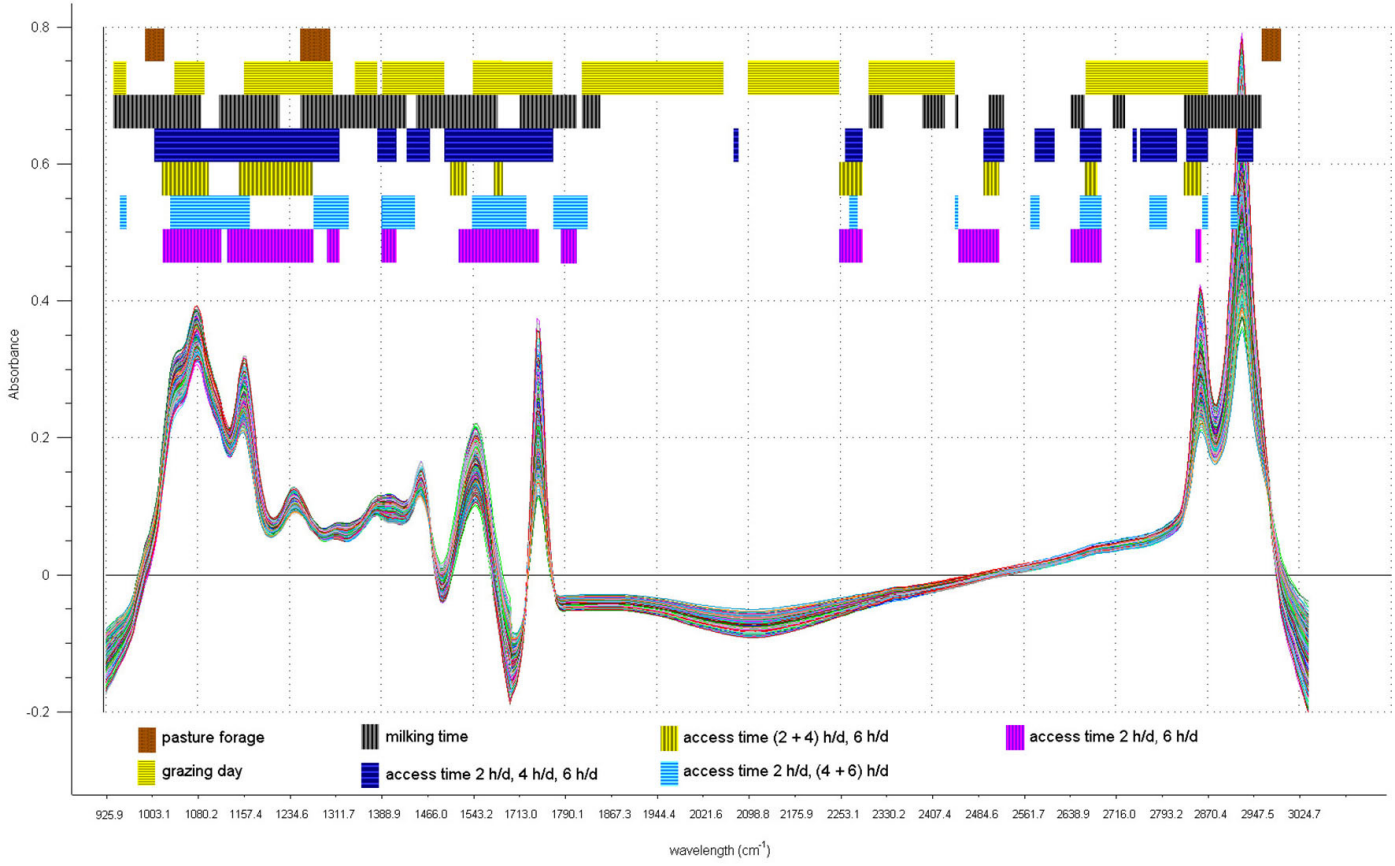
Spectral regions selected by genetic algorithm (GA) in all the GA-linear discriminant analysis (LDA) discrimination models.

The results expressed as percentages of correct classifications both in the training set (CV) and in the test set (prediction) are shown in Table 5, which also shows the spectral regions selected by GA for each discriminant model. The selected spectral regions are also shown in Figure 5.

**Table 5:** Percentage of correct classifications obtained by LDA using FT-MIR spectra as predictors.

**Predictors**	**Pasture forage**		**AT**		**AT**		**AT**		**AT**		**Grazing day**		**Milking time**	
	**Grass vs. Legume**		**2 vs. 4 vs. 6**		**(2+4) vs. 6**		**2 vs. (4+6)**		**2 vs. 6**		**Day 1 vs. Day 7**		**Morning vs. Afternoon**	
	**CV**	**Pred**.	**CV**	**Pred**.	**CV**	**Pred**.	**CV**	**Pred**.	**CV**	**Pred**.	**CV**	**Pred**.	**CV**	**Pred**.
PCA scores	78.9	76.0	38.5	39.8	55.1	55.0	54.7	56.1	55.0	55.6	55.2	56.4	91.5	91.0
Averaged spectra	100	100	56.4	60.5	70.1	70.8	71.2	75.6	73.0	78.5	92.6	93.7	98.5	98.6
GA selected regions	100	100	58.4	60.3	72.0	71.0	72.3	75.6	72.4	77.0	91.1	91.9	97.8	97.1
Selected spectral regions (cm^−1^)	995.4–1026.2 1261.6–1292.4 2962.9–2993.8		1006.9–1315.6 1377.3–1408.2 1435.2–1466.0 1493.0–1604.9 1701.4–1767.0 2071.7–2079.5 2256.9–2287.8 2488.4–2530.8 2581.0–2611.9 2650.4–2692.9 2743.0–2750.7 2766.2–2808.6 2835.6–2866.5 2928.2–2947.5		1018.5–1095.7 1145.8–1269.3 1504.6–1523.9 1574.1–1593.3 2245.4–2287.8 2395.8–2426.7 2662.0–2681.3 2824.1–2854.9		949.1–956.8 1030.1–1165.1 1273.1–1361.9 1388.9–1442.9 1539.3–1604.9 1701.4–1720.7 1770.8–1824.8 2268.5–2276.2 2442.1–2449.8 2476.8–2496.1 2557.8–2600.3 2777.8–2797.5 2858.8–2866.5 2916.6–2924.4		1018.51–1118.82 1134.25–1269.28 1284.71–1315.58 1388.88–1408.17 1516.2–1604.9 1701.4–1743.8 1782.4–1801.7 2245.4–2287.8 2453.7–2519.3 2638.9–2692.9 2847.2–2854.9		937.49–956.78 1041.66–1095.67 1157.40–1304.00 1342.58–1373.45 1388.88–1489.19 1539.34–1604.93 1701.38–1766.96 1817.12–2056.31 2094.89–2241.50 2303.23–2438.26 2662.02–2866.49		937.49–1084.10 1111.10–1211.41 1261.57–1431.32 1446.75–1581.78 1712.95–1801.69 1817.12–1847.98 2303.23–2322.52 2384.24–2426.68 2442.11–2449.83 2499.98–2530.85 2638.87–2658.16 2708.32–2727.61 2824.06–2959.09	
N. selected variables	27		222		102		153		168		324		258	
N. spectral regions	3		14		8		14		11		11		13	

*CV, cross validation; pred, prediction based on test data set; PCA, principal component analysis; GA, genetic algorithm*.

#### Discrimination of Pasture Forage

When discriminating the pasture forages, the model built using the PCA scores led to 78.9% of correct classification for the training set and to 76.0% of correct predictions of the external sample set, whereas 100% of samples were correctly classified in the calibration and in the validation steps, using either the whole averaged spectra or the variables selected by the GA (Table 5).

#### Discrimination of the AT to Pasture

The three AT categories (2 h/day vs. 4 h/day vs. 6 h/day) were poorly discriminated using the PCA scores as predictors, with only 38.5 and 39.8% of correct classifications for the training and the test sets, respectively. The results improved when using the whole averaged spectra as predictors reaching 56.4% and 60.5% of correct classifications for the two sample sets. The application of GA led to a reduction of variables from 525 to 222, corresponding to 14 spectral regions, but the model accuracies were not improved, with 58.4 and 60.3% of samples correctly predicted in the training and test sets, respectively.

When grouping the 2 h/day samples and the 4 h/day samples in the same category and comparing them to the 6 h/day samples, the discriminant model obtained using the PCA scores led to 55.1% of correct classification for the training set and to 55.0% of correct predictions for the test set. The use of the averaged spectra as predictors, improved the results to 70.1 and 70.8%, respectively. Using the GA-selected spectral variables led to similar results: 72.0 and 71.0% of correct classifications for the training and test sets, respectively.

Slightly better accuracies were achieved when the 4 h/day samples were grouped together with the 6 h/day samples in the same category and discriminated from the 2 h/day samples. The PCA scores as predictors led to 54.7 and 56.1% of calibration and validation accuracies. The corresponding values were 71.2 and 75.6%, respectively using the whole averaged spectra as predictors, and 72.3 and 75.6% with the GA-selected variables.

Finally, the discrimination of the 2 h/day samples from the 6 h/day samples did produce the best accuracy among the AT trials when using the whole averaged spectra or the GA-selected variables as predictors. In fact, the use of the PCA scores in the building of the model led to 55.0 and 55.6% of correct classifications in the samples of the training and test sets, respectively, but the use of the whole averaged spectra as predictors led to 73.0% of correct classifications in the calibration step and 78.5% of correct prediction in the validation step. The application of GA to the spectra reduced the number of variables used as predictors, from 525 to 168, and the model correctly predicted 72.4% of the training set samples and 77.0% of the test set samples.

#### Discrimination of the Grazing Day

The model built using the PCA scores was not satisfactory as only 55.2% of samples were assigned to the right category (Table 5). The model built considering the whole averaged spectra as predictors led to an improvement of the results, with 92.8% of correct classification in the calibration step and 93.7% of correct predictions in the validation step. GAs selected 324 variables out of 525, leading to percentages of correct classification of 91.1% for the calibration step and 91.9% for the validation step.

#### Discrimination of the Milking Time

In this case, even the model built using the PCA scores as predictors led to good results, as the percentage of correct classifications was 91.5% for the training set samples and 91.0% for the test set samples. The use of the whole averaged spectra led to 98.5 and 98.6% of correct classifications. The GA led to a reduction of about half of the total spectral variables (from 525 to 258), and to a correct classification of 97.8% of the training and 97.1% of the test samples, respectively.

## Discussion

### Univariate and PCA of Predicted FA Profile

Since the specific objective of this paper is the tracing of milk produced by ewes having different feeding regimes, we will briefly discuss the results of the above analysis, which set the benchmark for evaluating and interpreting the performance of the discriminant analysis. As expected, the univariate analysis of milk FA showed that increasing the amount and quality of herbage in the diet enhances the level of beneficial FA in milk (2). In fact, this study explores a wide range of diets, going from low quantity (2 h/day) of moderate quality herbage (day 7 on the grass) to high quantity (6 h/day) of high-quality herbage (day 1 on the legume, Table 1). In particular, the average intake of grass on the AT 2 h/day was 648 g DM (35% of total intake), with the neutral detergent fiber (NDF) level of 493 g/kg DM and a crude protein (CP) level of 119 g/kg DM on day 7 (3), whereas on the legume with AT of 6 h/day, the average intake was 1,723 g DM (62% of total intake), with the NDF level of 328 g/kg DM and CP level of 231 g/kg DM on day 1 (4). We can reasonably argue that this wide range of nutrient composition was mirrored by an even wider range of FA intake, since berseem clover has usually higher content of long-chain fatty acid (LCFA) than the Italian ryegrass, according to our laboratory data (19, 20). Although the grazed forage had a major impact on FA profile, since the supplementation changed between experiments, we cannot rule out that the different types of supplements can have an impact on the results obtained. For this reason, we conventionally refer to the effect of pasture forage combined with the supplementation type, nested in the trials (E1 and E2) as forage/trial effect.

It is well-known that leaves contain more LCFA than stems, as observed in berseem clover by Cabiddu et al. (20). This can suggest a higher intake of LCFA on the 1st day than on the last grazing day of the grazing periods throughout the experiments.

Actually, the effect of the different forages/trials was very strong on all variables. The effect of the grazing day was evident in most of them but the effect of AT was moderate, with a higher content of short FA and n-3 in the milk obtained from the highest AT (6 h/day), being 4 h/day intermediate. This is because the intake of herbage was also intermediate in these ewes as compared to the extreme levels of AT (3, 14). Moreover, the level of linoleic acid was possibly higher in the diets of sheep with lower AT, since lupin and maize are rich in linoleic acid (21, 22). This can explain why FA tended to be higher in milk samples from low-AT ewes.

Milking time has been so far an overlooked factor of milk FA composition in sheep. Few studies refer to the effects of milking times in grazing cows (19), but the milking schedule and feeding regimes are very different from the feeding background which is under scrutiny for a useful comparison. In our conditions, the schedule of grazing allocation in the morning and of supplementary main meals in the afternoon probably favored an increase of beneficial FA in the morning milk rather than in the afternoon milk, with exception of n-3. In the prevailing n-3 FA, linolenic acid was however not affected likewise. It is possible that other n-3 FAs, such as EPA and DHA were responsible for this inconsistency. In general, the longer n-3 needs several elongation and desaturation steps which may explain for a longer lag time between the intake of precursors and appearance in milk. Other long-chain PUFA can also be contained in lupin seed (22). In general, afternoon samples were characterized by higher levels of linoleic and oleic acid which can be sourced from supplements [linoleic acid in maize and lupin and oleic acid in lupin (21, 22), and/or the fat depot mobilization (oleic acid).

### Discriminant Analysis Using the FA Profile as Predictors

Pasture forages/trials were accurately discriminated using the FA profile and GA selected only six informative variables, such as C18:2 9c 12c, C18:3 9c 12c 15c, C18:2 9c 11t, PUFA, n-6, and n-3. All the above FAs, with the exception of the linoleic acid, were higher in the milk of legume-grazing ewes. Discrimination between different forage-based diets is rather uncommon using this LDA approach, but previous results of our laboratory showed that legumes tend to increase the level of C18:1 11t, C18:2 9c 11t, and occasionally C18:3 9c 12c 15c and n-6 in sheep milk (23).

In contrast, AT was rather poorly discriminated using the FA profile, for several reasons: first, this discrimination was more challenging since three categories were implied instead of two. Second, as shown by the univariate analysis, the difference between AT levels was evident only in a small number of FA, basically from *de novo* synthesis at the mammary gland level, with the exception of C4:0. Third, since there were three instead of two categories under focus, a smaller number of samples was present per category. The diet including a higher level of grazed herbage (6 h/day) was probably able to increase ruminal acetate production which is the main precursor for milk fat synthesis. However, since the supplementation partially compensated for the lower herbage intake in the ewes with the lowest AT (2 h/day), the GA-LDA of their FA profile poorly discriminated the 2 h/day from the 6 h/day samples, showing a large error (39 % of the test set samples). Interestingly, almost the same FAs were selected by GA in the four different trials that regarded AT to pasture discrimination. The common selected FAs were all the SFAs, except C14:0, C18:1 11t, UFA, and n-6. Distinguishing among mixed diets, including grazed herbage and supplements is a very challenging task, as already demonstrated by Coppa et al. (10).

The GA-LDA of the FA profile performed better when comparing milk from different grazing days. In fact, the milk collected in the first and last grazing days of the rotational scheme implemented in both studies was distinguished with 73.0% of accuracy in the test set. Interestingly, among the selected variables, some are also indicators of long AT, in particular, the short- and medium-chain FAs, such as C6:0, C8:0, C12:0, and C14:0. The long-chain FAs were selected and their classes are all probably related to the level of precursors in the herbages, such as C18:1 11t, PUFA, n-6, and n-3, being higher in the first than in the last grazing day, whereas C18:1 9c (an indicator of body fat mobilization but also present in lupin), C18:2 9c 12c (concentrated in maize grain), and SFA were all related to high supplementation of proportion in the diet and poor herbage precursor intake and uptake.

The GA-LDA of FA profile was able to classify samples collected at different milking times with good accuracy (88.8% of the test set samples). This is in line with the relevant effect of milking time in the univariate analysis. Our milking schedule was thoroughly abided with an 8 h interval between morning and afternoon milking and a 16 h interval between afternoon and morning milking. This can explain why milking time affected most of the milk FA, consistently across studies, with a few exceptions. Some of the GA-selected FA and FA classes were higher in the morning samples (C6:0, C8:0, C10:0, C14:0, C16:0, C18:2 9c 11t, SFA, PUFA, and n-3, Table 4), mostly mirroring the intake of herbage precursors (except for SFA). The other GA-selected FA and FA classes were higher in the afternoon samples (C18:1 9c, C18:2 9c 12c, UFA, and n-6; Table 4) mostly mirroring the supplementation regime and the energy balance. To our knowledge, such a discrimination approach has not been implemented so far.

### Discriminant Analysis Using the FT-MIR Spectra as Predictors

Overall, the models built using FT-MIR of milk spectra (Table 5) gave better prediction accuracy than those based on FT-MIR of FA profile (Table 4). This advantage of spectra is explainable by the prediction error of FA (16), although small, which obviously does not affect the spectra. Moreover, spectra contain information that goes far beyond FA composition, being also related to other milk components and the interaction between them.

Focusing on spectra-based LDA (Table 5), the predictions obtained by the PCA score-LDA models were overall less accurate than those obtained by the averaged spectra-LDA models and the GA-LDA models. The difference between LDA of averaged spectra and GA-LDA of whole spectra were minimal but the models built with a selection of variables are to be preferred as they contain only the informative variables. This makes these parsimonious models more simple and less sensitive to random variability, and therefore more stable and reliable for future predictions.

Despite the benefits of LDA of milk spectra as compared with that of predicted FA profile, spectra are *per se* less interpretable than predicted FA content, unless we are able to relate the absorbance at specific wavelengths with the presence/content of FA in milk. The following sections are devoted to this aim.

In the discrimination of the pasture forage, a connection between the FAs and the spectral regions selected by GA was found, since the region from 995.4 to 1026.2 cm^−1^ is related to the absorbance of the C-H group bound to double bonds in *trans* configuration, present in C18:1 11t and C18:2 9c 11t. The latter FA was also selected by the GA-LDA of the FA profile. Actually, C18:1 11t and C18:2 9c 11t were much higher in the milk sampled from the legume than the grass-grazing ewes. In contrast, the regions from 1261.6 to 1292.4 cm^−1^ and from 2962.9 to 2993.8 cm^−1^ have no apparent connection to the selected FAs. In fact, these regions have no typical absorbance of chemical groups that differ in the FAs. For example, the last region is typical of the stretching vibrations of C-H bond in methyl groups which are present in all milk FA.

When discriminating the three AT categories, the GA selected only three regions that could contain absorbance peaks due to vibrations of chemical bonds present in FAs, and in particular, to the bending and stretching vibrations of C-H bond in methylene groups, present in saturated carbon chains; these regions are 1435.2 to 1466.0 cm^−1^, 2835.6 to 2866.5 cm^−1^, and 2928.2 to 2947.5 cm^−1^. This is partially in line with the selection by GA of C4:0, C6:0, C8:0, C10:0, C12:0, C16:0, and C18:0 in the LDA based on the FA profile.

Similar spectral regions were selected for distinguishing the 2 h/day from the 4 and 6 h/day samples: from 1388.9 to 1442.9 cm^−1^, from 2858.8 to 2866.5 cm^−1^, and from 2916.6 to 2924.4 cm^−1^. These regions contain wavelengths on which the C-H bond in methylene groups absorbs the infrared beam light.

Instead, none of the spectral regions selected to discriminate the 6 h/day from the 2 and 4 h/day samples and the 2 h/day from the 6 h/day samples are related to any chemical bond that differs in the types of FAs and classes of FAs present in milk.

When discriminating for the grazing day, GA selected 11 spectral regions, two of which are related to saturated carbon chains, and consequently to the amount of sum of SFAs, which was also selected by the GA-LDA of FA profile.

The spectral regions selected by GA for the discrimination of different milking times contain absorbance peaks related to the bending vibrations of the C-H bond in *trans*-configuration double C=C bond (937.5 to 1084.1 cm^−1^) and to the bending and stretching vibrations of the C-H bond in methylene groups (from 1446.7 to 1581.8 cm^−1^ and from 2824.1 to 2959.1 cm^−1^, respectively). The first region could be therefore related to the amount of C18:2 9c 11t (one of the FAs selected by GA in the LDA of FA profile), whereas the latter two regions could be related to the amount of other selected FA, such as SFA and the individual SFAs, such as C6:0, C8:0, C10:0, C14:0, and C16:0. All the other selected spectral regions are not directly related to any selected fatty acid.

To sum up the discussion on the GA-LDA of FA and spectra, it is worthy to note that in the discrimination of the pasture forages/trials, AT, grazing days, and milking times in the GA-LDA based on FA, some of the selected FAs were common (C18:1 9c, C18:2 9c 12c, PUFA, n-6, and n-3, Table 4). Likewise, in the GA-LDA based on FT-MIR spectra, some of the selected regions were common (Figure 5). This recalls the gradient of precursor and nutrient intake that was explored in this study. In fact, the above FAs are indicators of herbage intake, and C18:1 9c, in particular, can also be sensitive to energy balance. Milk from ewes grazing only for 2 h/day were in fact the ones that show lower contents of some beneficial FAs, such as n-3, but also a higher content of oleic acid, possibly related to a higher desaturation of C18:0 at the fat tissue level.

## Conclusions

The comparison between the performance of the multivariate models confirms that the models using the GA-selected variables are to be preferred, as only the informative variables are retained, making the predictions more robust and hence reliable to be implemented to external data sets. The discrimination performance of GA-LDA as expected was better when the spectra were used instead of the milk FA content, estimated on the basis of previously validated calibrations, although the difference in accuracy between the approaches varied among targeted comparisons.

Individual milk samples from ewes under a rotational PTG of *Lolium multiflorum* and *Trifolium alexandrinum* were well-discriminated using the GA-LDA of their FA profile and even better applied using the same statistic to their FT-MIR spectra.

However, GA-LDA based only on FT-MIR spectra discriminated accurately individual milk samples collected in the first grazing day from those collected in the last grazing day and those collected in the morning from those collected in the afternoon milking.

In contrast, neither the GA-LDA of FA nor GA-LDA of spectra were able to accurately disentangle samples obtained from ewes having 2, 4, or 6 h/day AT to pasture, although the error was limited to c.a. 25% of samples with GA-LDA of spectra, if only the extreme AT milk were compared. This is in line with univariate analysis results which showed differences only for a few FA, between milk sourced from ewes with 2 and 6 h/day of AT to pasture.

These findings overall suggest that the best GA implemented in this study (GA-LDA of FT-MIR spectra) provides encouraging results for discriminating morning vs. afternoon milk samples and for tracing individual sheep milk back to sheep feeding regimen, with reference to the grazed forage and the grazing day, which can be regarded as an indicator of quality/amount of herbage eaten in rotationally stocked sheep. On the contrary, results are not yet fully satisfactory when discriminating mixed diets of ewes, part-time grazing with AT to pasture differing by 2 or 4 h/day.

## Data Availability Statement

The raw data supporting the conclusions of this article will be made available by the authors, without undue reservation.

## Ethics Statement

The animal study were reviewed and approved by Agris Ethical committee.

## Author Contributions

GM, AC, MD, and VG conceived the idea and performed the experimental design. MS helped for the field experiment. II carried out the milk chemical analysis. MA and MC carried out the simulation in the implementation of milk fatty acid analysis and spectral by chemometric approach. GM supervised the project. All authors discussed the results and contributed to the final manuscript.

## Conflict of Interest

The authors declare that the research was conducted in the absence of any commercial or financial relationships that could be construed as a potential conflict of interest.
